# Comparative efficacy of antidepressants for acute adolescent major depressive disorder: a source-verified systematic review and network meta-analysis

**DOI:** 10.3389/fpsyt.2026.1881200

**Published:** 2026-08-28

**Authors:** Chan Gong, Wengao Zeng, Xiangzhi Xiao, Qing Liu, Yaoyue Luo

**Affiliations:** 1 School of Nursing, Hunan University of Chinese Medicine, Changsha, Hunan, China; 2 Department of Internal Medicine, The Second Hospital of Changsha (West Branch of Changsha Hospital for Maternal and Child Health Care), Changsha, Hunan, China

**Keywords:** adolescent major depressive disorder, antidepressants, evidence robustness, network meta-analysis, reporting integrity, source verification

## Abstract

**Background:**

Adolescent-specific evidence on antidepressants is sparse and difficult to interpret because outcome data structures are heterogeneous, safety reporting is incomplete, and post-publication reanalyses have identified major reporting-integrity problems in influential trials. We aimed to estimate the comparative acute efficacy of antidepressants using source-verifiable, adolescent-specific arm-level endpoint data and to examine how historical trials with important data-quality limitations influenced the estimates.

**Methods:**

We searched PubMed/MEDLINE, Cochrane CENTRAL, Web of Science, and ClinicalTrials.gov from inception to April 2026 for randomized controlled trials of antidepressants for acute treatment of adolescents aged 12–18 years, or trial-defined samples aged 12–17 or 13–18 years, with a primary DSM/ICD diagnosis of MDD. Eligibility for the primary efficacy analysis required source-verifiable, adolescent-specific arm-level endpoint means, standard deviations, and sample sizes at 12 weeks or earlier; pill placebo served as the network reference. Continuous depression outcomes were expressed as Hedges’ g standardized mean differences (SMDs), placing eligible validated scales on a common metric. Risk of bias was assessed with RoB 2, and influence analyses examined robustness to the exclusion of historical trials with major data-quality limitations.

**Results:**

The antidepressant-only primary SMD network contained 4 parent trials and 11 randomized arms, including desipramine, amitriptyline, fluoxetine, levomilnacipran 40 mg, levomilnacipran 80 mg, vilazodone 15 mg, vilazodone 30 mg, and pill placebo. Desipramine showed a large effect versus pill placebo (SMD -1.80, 95% CI -2.57 to -1.03), but this estimate came from a small historical trial using completer endpoint data and a reported pooled/common SD. Amitriptyline (-0.17, -0.88 to 0.55), fluoxetine (-0.15, -0.39 to 0.08), levomilnacipran 80 mg (-0.15, -0.38 to 0.09), vilazodone 30 mg (-0.12, -0.33 to 0.09), levomilnacipran 40 mg (-0.11, -0.35 to 0.13), and vilazodone 15 mg (-0.02, -0.23 to 0.19) did not differ significantly from pill placebo. After excluding Klein 1998, and again after excluding both Klein 1998 and Kye 1996, no remaining antidepressant showed a statistically significant advantage over pill placebo.

**Conclusions:**

Contemporary antidepressants in the primary network (the selective serotonin reuptake inhibitor [SSRI] fluoxetine, the serotonin-norepinephrine reuptake inhibitor [SNRI] levomilnacipran, and vilazodone, an SSRI and 5-HT1A receptor partial agonist) did not show statistically significant superiority over pill placebo, although the confidence intervals remained compatible with small benefit as well as no effect. The only statistically significant estimate was derived from a small historical desipramine trial with important data-quality limitations. Adolescent-specific arm-level evidence was too sparse and methodologically limited to support a stable comparative efficacy hierarchy among antidepressants.

## Introduction

Major depressive disorder (MDD) in adolescence is associated with psychosocial impairment, educational disruption, recurrence, and elevated suicide risk (1). Recent global evidence indicates a high burden of adolescent depression and depressive symptoms, reinforcing the need for treatment decisions that are both evidence-based and appropriately cautious (2, 3).

Guidelines generally recommend evidence-based psychological interventions for mild to moderate depression and careful consideration of pharmacotherapy, particularly fluoxetine, for moderate to severe depression or when psychological treatment is insufficient (4, 5). However, the comparative evidence for adolescent MDD remains difficult to interpret because trials vary in diagnostic strictness, age range, outcome scales, treatment duration, reporting completeness, and adverse-event ascertainment.

Earlier NMAs provided important benchmarks. Cipriani et al. evaluated antidepressants in children and adolescents with MDD (6), and Zhou et al. integrated antidepressants, psychotherapies, and combined treatments across children and adolescents with depressive disorders (7). Their broader age ranges, mixed intervention classes, and heterogeneous outcome structures leave uncertainty about the comparative acute efficacy of antidepressants when evidence is limited to adolescent-specific, source-verifiable arm-level endpoints.

We aimed to estimate the comparative acute efficacy of antidepressants for adolescent MDD using source-verifiable, adolescent-specific arm-level endpoint data. We also examined the robustness of the estimates to the exclusion of historical trials with major data-quality limitations.

## Methods

This systematic review and NMA was reported in accordance with the PRISMA 2020 statement and the PRISMA extension for NMA (8, 9). Study eligibility, data extraction, and synthesis were defined according to population, antidepressant intervention, comparator, acute endpoint timing, outcome data structure, source verifiability, and network connectivity. Risk of bias was assessed using the revised Cochrane risk-of-bias tool for randomized trials (RoB 2) (10).

### Protocol and registration

The review was not prospectively registered in PROSPERO. Eligibility criteria and study-classification workbook, source-verification records, RoB 2 assessments, source-verified study-characteristics table, analysis datasets, and analysis R scripts are provided in the **Supplementary Material**.

### Search strategy and study selection

Electronic searches were conducted in PubMed/MEDLINE, Cochrane CENTRAL, Web of Science, and ClinicalTrials.gov from database inception to April 2026. The strategy combined terms for adolescents or youth, major depressive disorder or depression, randomized or controlled trials, and pharmacological or psychological interventions.

Records retrieved from electronic sources were de-duplicated before two-stage screening. Titles and abstracts were screened first, and potentially eligible reports were assessed in full text against the eligibility criteria. Multiple reports from the same parent trial were collated. For potentially eligible antidepressant trials, source availability, population and diagnostic eligibility, endpoint timing, outcome-data structure, and network connectivity were verified before quantitative synthesis.

### Eligibility criteria

Eligible studies were randomized controlled trials evaluating an antidepressant during acute treatment of adolescent MDD, with an endpoint at 12 weeks or earlier. Eligibility criteria were defined according to the PICOS framework. The population comprised adolescents aged 12–18 years, or trial-defined adolescent samples aged 12–17 or 13–18 years, with a primary DSM or ICD diagnosis of MDD. Comparators were pill placebo or another antidepressant. Eligible outcomes included acute-phase depressive-symptom severity, response or remission, acceptability, and safety.

For quantitative synthesis of the primary continuous outcome, comparisons were eligible when adolescent-specific arm-level endpoint means, standard deviations, and sample sizes could be verified from source reports. When multiple eligible time points were available, we preferentially extracted the prespecified primary acute endpoint or the time point closest to 8–12 weeks. Mixed child-adolescent or adolescent-adult trials were eligible only when adolescent-specific arm-level data could be extracted without disrupting the randomized comparison.

We excluded non-randomized studies, observational studies, single-arm studies, protocols, reviews, maintenance or relapse-prevention trials, open-label extension phases without eligible acute randomized data, and studies without extractable data. Studies were also excluded from the primary analysis when MDD could not be separated from dysthymia, depressive disorder not otherwise specified, non-clinical depressive symptoms, bipolar depression, psychotic depression, or treatment-resistant depression-focused samples.

### Data extraction

One investigator performed the initial data extraction, and a second investigator checked all primary-analysis arm-level data against source pages. Multiple reports from the same parent trial were collated. Secondary publications, trial registries, company reports, regulatory documents, and post-publication reanalyzes were used to verify trial identity, supplement outcome information, and assess reporting integrity.

Extracted continuous-outcome data included treatment arm, sample size, depression scale, endpoint timing, endpoint mean, standard deviation, analysis population, and source location. Adjusted least-squares means, standard errors, model-based contrasts, and binary response or remission data were recorded separately and were not combined with source-verified arm-level endpoint mean/SD data. Effect measures and synthesis methods were matched to the outcome data structure in accordance with Cochrane guidance (11).

### Outcomes

The primary efficacy outcome was depressive symptom severity at the acute endpoint. The primary effect measure was Hedges’ g standardized mean difference (SMD), which expresses eligible continuous depression outcomes on a common standardized metric (11). Negative values indicate lower endpoint depressive-symptom scores and therefore favor active treatment. A CDRS-R-only MD analysis was performed as a clinical-unit sensitivity analysis.

Binary response or remission outcomes were summarized as direct evidence and were not combined with the continuous-outcome network. Safety outcomes and post-publication concerns about selective reporting were summarized narratively (12–18).

### Statistical analysis

Analyses were conducted in R version 4.5.3 using the netmeta package (19). Treatment effects were summarized as Hedges’ g standardized mean differences (SMDs) with 95% confidence intervals (CIs), placing eligible validated depression scales on a common metric. Pill placebo was specified as the reference treatment, and random-effects network estimates were calculated for eligible antidepressant comparisons in the connected component containing that reference. A CDRS-R-only mean-difference sensitivity analysis and influence analyses excluding Klein 1998 and Kye 1996 were conducted. Reported or model-derived least-squares mean differences, adjusted estimates, and binary outcomes were summarized separately.

### Source verification, risk-of-bias, and data-structure assessment

Each arm-level value used in the SMD network was traced to a source page and table or results paragraph. Data-quality indicators were recorded for completer endpoints, reported pooled/common SDs, model-derived estimates, and ITT-versus-completer distinctions. Risk of bias was assessed using RoB 2 across five domains: the randomization process, deviations from intended interventions, missing outcome data, outcome measurement, and selection of the reported result (10). Source availability, endpoint scale and timing, analysis population, missing-data handling, estimate type, arm-level data availability, post-publication reporting concerns, and denominator definitions were recorded alongside the RoB 2 judgments. RIAT reanalyses informed the selection-of-the-reported-result judgments and the assessment of reporting integrity.

## Results

### Study selection and network characteristics

The search identified 4,351 records. After duplicate removal, 4,249 records were screened by title and abstract, and 321 full-text articles were assessed. Four parent trials comprising 11 randomized arms met the criteria for the primary NMA. The study-selection process is shown in **Supplementary Figure 1**, and trial classifications are summarized in **Supplementary Table 1**.

The source-verified primary antidepressant endpoint mean/SD SMD network comprised 4 parent trials and 11 randomized arms. Depressive symptom outcomes in this network were measured using either the Children’s Depression Rating Scale-Revised (CDRS-R) or the Hamilton Depression Rating Scale (HAM-D). The four parent trials were Klein 1998, Kye 1996, Durgam 2018, and Radecki 2024 LVM-MD-11 (20–23). The included treatment nodes were desipramine, amitriptyline, fluoxetine, levomilnacipran 40 mg, levomilnacipran 80 mg, vilazodone 15 mg, vilazodone 30 mg, and pill placebo. The network therefore included two tricyclic antidepressants (TCAs; desipramine and amitriptyline) and three types of contemporary antidepressant: an SSRI (fluoxetine), an SNRI (levomilnacipran), and an SSRI with 5-HT1A receptor partial agonist activity (vilazodone). The primary antidepressant network is shown in **Supplementary Figure 2**, and arm-level source verification for that network is provided in **Supplementary Table 2**.

### Primary SMD NMA

**Figure 1:**
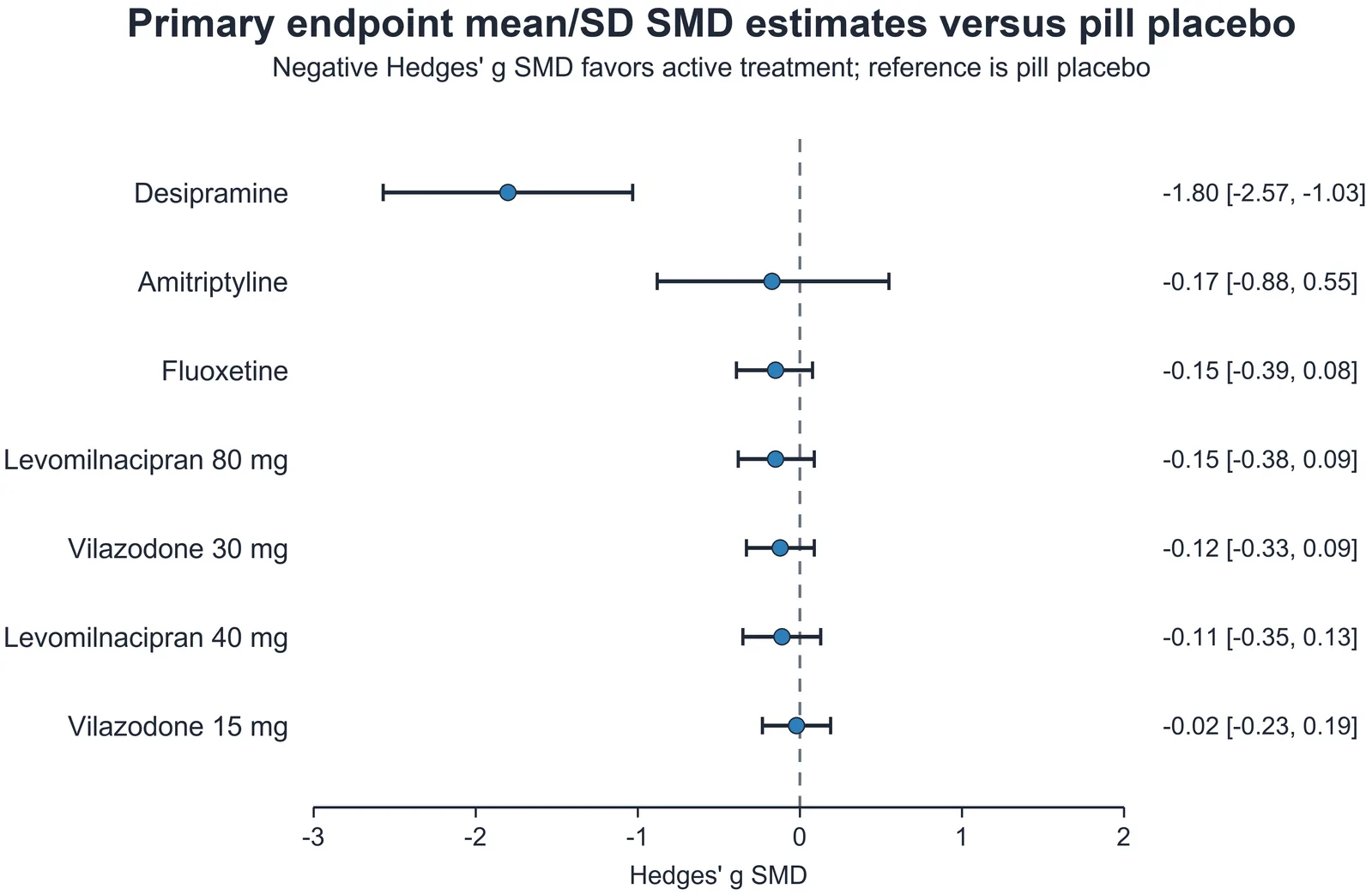
Random-effects estimates from the antidepressant endpoint mean/SD SMD network versus pill placebo. Negative Hedges’ g values favor active treatment. Desipramine was informed by a small historical trial using completer endpoint data and a reported pooled/common SD.

In the primary SMD NMA, desipramine showed a statistically significant advantage over pill placebo (SMD -1.80, 95% CI -2.57 to -1.03). The contributing trial used completer endpoint data and a reported pooled/common SD. Confidence intervals crossed the null for amitriptyline (SMD -0.17, 95% CI -0.88 to 0.55), fluoxetine (-0.15, -0.39 to 0.08), levomilnacipran 80 mg (-0.15, -0.38 to 0.09), vilazodone 30 mg (-0.12, -0.33 to 0.09), levomilnacipran 40 mg (-0.11, -0.35 to 0.13), and vilazodone 15 mg (-0.02, -0.23 to 0.19) (**Figure 1**; **Table 1**).

**Table 1:** Primary source-verified antidepressant SMD NMA estimates versus pill placebo.

Treatment	Hedges’ g SMD	95% CI	p value	Interpretation note
Desipramine	-1.80	-2.57 to -1.03	<0.001	TCA; small historical trial; completer endpoint; reported pooled/common SD
Amitriptyline	-0.17	-0.88 to 0.55	0.643	TCA; historical trial; ITT/protocol-end endpoint flag
Fluoxetine	-0.15	-0.39 to 0.08	0.207	Contemporary SSRI; placebo-connected trial
Levomilnacipran 80 mg	-0.15	-0.38 to 0.09	0.226	Contemporary SNRI; placebo-connected trial
Vilazodone 30 mg	-0.12	-0.33 to 0.09	0.248	Contemporary SSRI and 5-HT1A receptor partial agonist; placebo-connected trial
Levomilnacipran 40 mg	-0.11	-0.35 to 0.13	0.367	Contemporary SNRI; placebo-connected trial
Vilazodone 15 mg	-0.02	-0.23 to 0.19	0.879	Contemporary SSRI and 5-HT1A receptor partial agonist; placebo-connected trial

Negative SMD values favor active treatment. SSRI, selective serotonin reuptake inhibitor; SNRI, serotonin-norepinephrine reuptake inhibitor; TCA, tricyclic antidepressant.

### Robustness to historical trials evaluating TCAs

**Figure 2:**
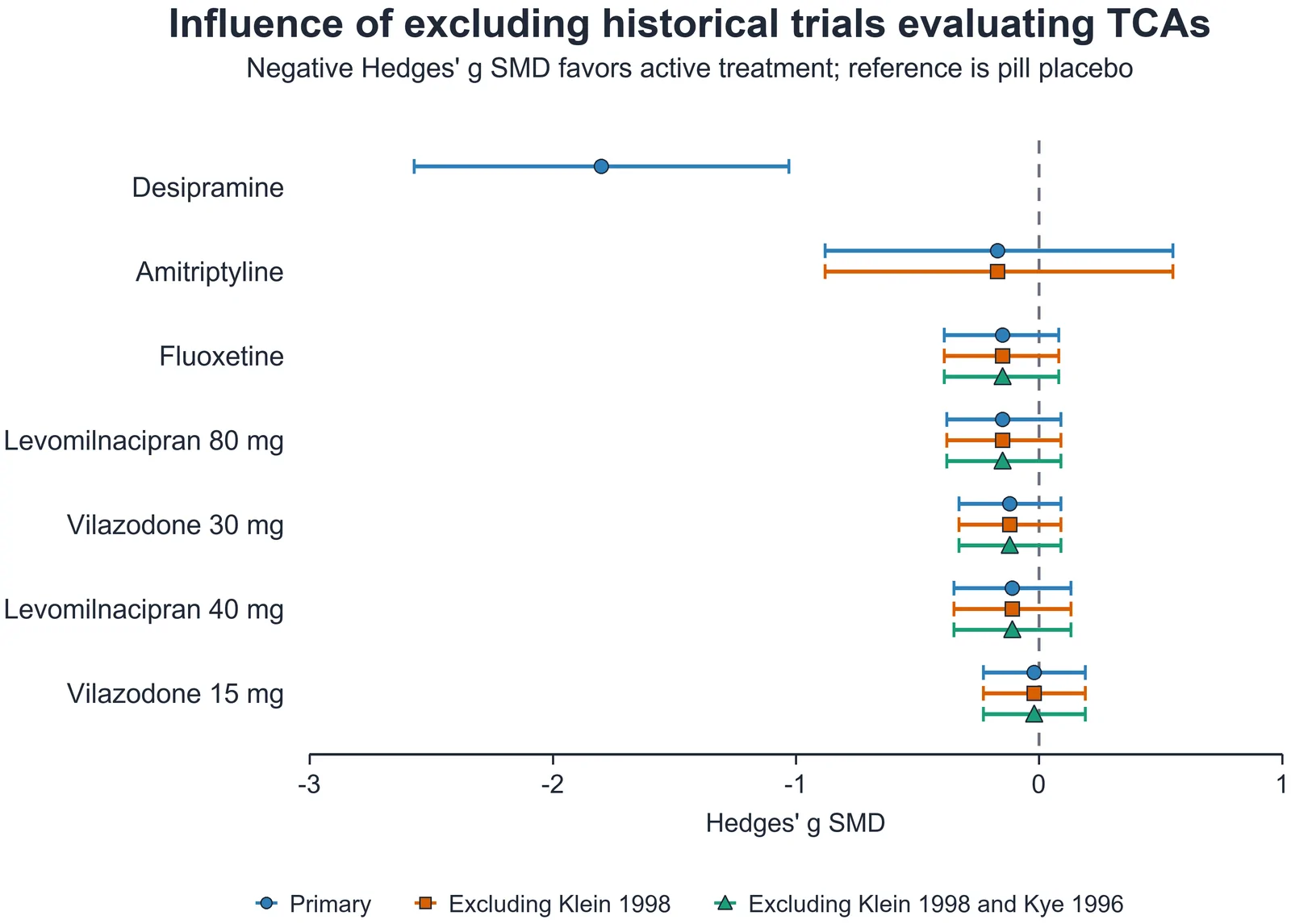
Influence of historical trials evaluating TCAs on antidepressant estimates. Excluding Klein 1998 removed desipramine from the network; excluding both Klein 1998 and Kye 1996 left only contemporary placebo-connected antidepressant trials, with no statistically significant estimate versus pill placebo.

After excluding Klein 1998, desipramine was absent from the network and no remaining treatment differed significantly from pill placebo. After excluding both Klein 1998 and Kye 1996, the network contained Durgam 2018 and Radecki 2024 LVM-MD-11 only; fluoxetine, levomilnacipran 40 mg, levomilnacipran 80 mg, vilazodone 15 mg, and vilazodone 30 mg remained statistically non-significant versus pill placebo (**Figure 2**; **Supplementary Table 3**).

### Sensitivity analyses

In the CDRS-R-only connected MD sensitivity analysis, estimates were numerically favorable but not statistically significant for fluoxetine (MD -2.10, 95% CI -5.59 to 1.39), levomilnacipran 80 mg (-2.00, -5.30 to 1.30), levomilnacipran 40 mg (-1.50, -4.81 to 1.81), vilazodone 30 mg (-1.50, -4.07 to 1.07), and vilazodone 15 mg (-0.20, -2.83 to 2.43). The connected and disconnected CDRS-R-only MD results are shown in **Supplementary Figures 3** and **4**.

Model-based active-versus-placebo contrasts were available for fluoxetine, escitalopram, paroxetine, imipramine, levomilnacipran, and vortioxetine. Estimates favored fluoxetine in Findling 2022 and escitalopram in Emslie 2009; estimates for paroxetine, imipramine, levomilnacipran, and vortioxetine were imprecise or not statistically significant (12, 24–26). These results are shown in **Supplementary Figure 5** and **Supplementary Table 4**.

Binary response or remission outcomes were reported in two parent trials. Direct estimates are shown in **Supplementary Figure 6** and **Supplementary Table 5**.

### Source verification and data-quality flags

Source-page verification showed that Durgam 2018 contributed observed week-8 CDRS-R endpoint mean/SD values rather than LS mean changes, and that Radecki 2024 LVM-MD-11 contributed observed week-8 endpoint mean/SD values, with LSM/SE values recorded separately (22, 23). Klein 1998 had a completer-endpoint and reported pooled/common-SD flag, and Kye 1996 had an ITT/protocol-end endpoint flag (**Table 2**; **Supplementary Tables 2**, **6**) (20, 21).

**Table 2:** Primary arm-level source verification summary.

Trial	Treatment arm	n	Scale and endpoint	Endpoint mean (SD)	Source	Data-quality note
Klein 1998	Desipramine	18	HAM-D, week 6	10.83 (2.10)	pp. 10-11, **Table 6**	Completer endpoint; reported common pooled SD.
Klein 1998	Pill placebo	18	HAM-D, week 6	14.61 (2.10)	pp. 10-11, **Table 6**	Completer endpoint; reported common pooled SD.
Kye 1996	Amitriptyline	18	HAM-D, week 8	8.00 (4.90)	pp. 3-4, Results; **Table 2**	Reported ITT protocol-end endpoint; manual ITT/completer flag retained.
Kye 1996	Pill placebo	13	HAM-D, week 8	8.80 (4.50)	pp. 3-4, Results; **Table 2**	Reported ITT protocol-end endpoint; manual ITT/completer flag retained.
Durgam 2018	Vilazodone 15 mg	174	CDRS-R, week 8	33.80 (12.00)	p. 7, **Table 2**	ITT observed week-8 endpoint; not an LSM change.
Durgam 2018	Vilazodone 30 mg	180	CDRS-R, week 8	32.50 (11.50)	p. 7, **Table 2**	ITT observed week-8 endpoint; not an LSM change.
Durgam 2018	Pill placebo	170	CDRS-R, week 8	34.00 (12.90)	p. 7, **Table 2**	ITT observed week-8 endpoint; not an LSM change.
Radecki 2024 LVM-MD-11	Levomilnacipran 40 mg	134	CDRS-R, week 8	38.10 (12.60)	p. 6, **Table 2**	Observed week-8 endpoint; LSM/SE data kept separate.
Radecki 2024 LVM-MD-11	Levomilnacipran 80 mg	138	CDRS-R, week 8	37.60 (12.70)	p. 6, **Table 2**	Observed week-8 endpoint; LSM/SE data kept separate.
Radecki 2024 LVM-MD-11	Fluoxetine	134	CDRS-R, week 8	37.50 (14.20)	p. 6, **Table 2**	Observed week-8 endpoint; LSM/SE data kept separate.
Radecki 2024 LVM-MD-11	Pill placebo	140	CDRS-R, week 8	39.60 (15.30)	p. 6, **Table 2**	Observed week-8 endpoint; LSM/SE data kept separate.

Values are endpoint mean (SD); lower HAM-D and CDRS-R scores indicate fewer depressive symptoms. p., page; pp., pages. HAM-D, Hamilton Depression Rating Scale; CDRS-R, Children’s Depression Rating Scale-Revised; ITT, intention-to-treat; LSM, least-square mean; SE, standard error. Full source-page details and full data-quality flags are provided in **Supplementary Table 2**. Demographic or safety denominators reported in **Supplementary Table 7** may differ from endpoint or ITT denominators used in this source-verification table; endpoint denominators were used for the primary SMD analysis.

### Risk-of-bias assessment

RoB 2 assessments are summarized in **Supplementary Table 6**. Among trials contributing to the primary SMD network, Klein 1998 was judged at high risk of bias, driven mainly by completer endpoint data in a small sample and the reported pooled/common SD; Kye 1996 was judged as having some concerns because randomization details, missing-data handling, and the ITT/protocol-end distinction were incompletely reported by current standards. Durgam 2018 and Radecki 2024 LVM-MD-11 were judged as having some concerns overall: both were contemporary randomized double-blind placebo-controlled trials with low-risk judgments for randomization, deviations from intended interventions, and outcome measurement, but missing-data assumptions could not be reanalyzed from aggregate data and selective-reporting concerns could not be fully excluded. Among trials represented in the supplementary model-based analyses, Keller 2001/Study 329 was classified as high risk because of major post-publication RIAT and selective-reporting concerns (**Supplementary Table 6**).

## Discussion

Using adolescent-only, acute-phase, source-verifiable endpoint mean/SD criteria, the primary antidepressant network included 4 parent trials and 11 randomized arms. Contemporary antidepressants in the primary network (the SSRI fluoxetine, the SNRI levomilnacipran, and vilazodone, an SSRI and 5-HT1A receptor partial agonist) did not show statistically significant superiority over pill placebo. The only statistically significant estimate was observed for desipramine (a tricyclic antidepressant), but it originated from a small historical trial with completer endpoint data and a reported pooled/common SD. This finding was not robust after excluding Klein 1998.

Compared with previous NMAs, the estimates in this study were more conservative. Cipriani et al. synthesized antidepressant trials across children and adolescents with MDD, whereas Zhou et al. integrated pharmacological, psychological, and combined treatments across broader child-adolescent depressive-disorder populations (6, 7). Differences in age range, diagnostic spectrum, intervention class, outcome definition, data structure, and network connectivity may account for the divergent estimates. The present analysis used adolescent-specific acute randomized evidence and did not combine source-verified arm-level endpoint mean/SD data with model-based estimates, binary outcomes, or disconnected components.

Broader mixed networks can incorporate indirect evidence from child-adolescent samples, combined-treatment trials, psychotherapy comparisons, model-based contrasts, or binary response/remission outcomes. In this NMA, most estimates for contemporary antidepressants (fluoxetine, levomilnacipran, and vilazodone) were informed by individual placebo-controlled trials, and their confidence intervals crossed the null. This should not be interpreted as evidence of no clinical benefit. Rather, the available adolescent-specific arm-level endpoint data were insufficient to demonstrate robust superiority over pill placebo.

The CDRS-R-only analysis provides a complementary clinical-unit perspective. The primary SMD analysis was appropriate for combining HAM-D and CDRS-R endpoint scores on a common standardized scale, whereas the CDRS-R-only MD analysis retained the original clinical scale but necessarily excluded HAM-D trials. The directionally favorable but imprecise CDRS-R-only estimates for these contemporary antidepressants are consistent with broader reviews showing small average symptom differences and low or very low certainty for many pediatric antidepressant comparisons (27). These findings suggest that the main uncertainty is not merely statistical power, but also the limited availability of adolescent-specific, source-verifiable, and consistently reported endpoint data.

The result for desipramine (a tricyclic antidepressant) illustrates the limitations of interpreting treatment hierarchy without considering trial era and data quality. Although desipramine appeared statistically favorable in the primary network, the estimate was generated by a small historical trial and was removed when Klein 1998 was excluded. Amitriptyline, another TCA, was not statistically superior to pill placebo. These findings do not support a clinical preference for TCAs in adolescent MDD; rather, they show that a treatment ranking can be disproportionately influenced by a single historical trial when the connected network is sparse.

Post-publication RIAT reanalyses documented serious discrepancies between the original published reports and analyses based on the underlying trial records. For Study 329, paroxetine and high-dose imipramine were neither statistically nor clinically superior to placebo on the prespecified efficacy outcomes, while harms increased, including suicidal ideation and behavior and other serious adverse events (12). For TADS, the RIAT reanalysis reported that fluoxetine monotherapy did not demonstrate superiority to pill placebo and identified 11 additional suicide-related adverse events that had not been reported in the original publications (13). Selective outcome reporting, outcome switching, and incomplete or misleading classification and reporting of harms compromised the integrity of the published evidence and materially affected interpretation of efficacy and safety (12, 13, 26, 28).

Clinical interpretation should emphasize the magnitude and precision of placebo-referenced effects, source verifiability, and trial-level data-quality limitations rather than a single treatment hierarchy. In adolescent MDD, placebo response, developmental heterogeneity, adverse-event ascertainment, and incomplete reporting may affect apparent antidepressant performance.

The RoB 2 assessment reinforces this interpretation. The primary network did not contain a trial with an overall low-risk RoB 2 judgment: the statistically significant desipramine estimate came from a high-risk historical trial, whereas Kye 1996 and the contemporary placebo-connected antidepressant trials were judged as having some concerns. Therefore, the main conclusion should not be framed as a definitive absence of antidepressant efficacy, but as a limitation of the available adolescent-only, source-verifiable, placebo-connected evidence. Conversely, the desipramine signal should not be interpreted as a clinically stable or hierarchy-defining advantage because it was both statistically influential and at high risk of bias.

## Limitations

Several limitations should be considered. First, the antidepressant-focused primary estimand and eligibility rules were defined *post hoc*, and the review was not prospectively registered; the analyses should therefore be considered exploratory. Second, the primary network was small and several estimates were informed by single parent trials, limiting precision and meaningful assessment of heterogeneity or incoherence. Third, the RoB 2 assessment identified important trial-level limitations: the statistically significant desipramine estimate came from a small historical trial at high risk of bias, while the contemporary placebo-connected antidepressant trials were judged as having some concerns. Fourth, some potentially relevant antidepressant trials were not synthesized in the primary network because they lacked extractable adolescent-specific arm-level endpoint mean/SD data, reported only model-based contrasts, or assessed endpoints beyond 12 weeks. Fifth, safety outcomes were summarized narratively because adverse-event and suicidality definitions, ascertainment, classification, and reporting were heterogeneous, and the RIAT reanalyses showed that influential publications omitted or misrepresented clinically important efficacy and harm information (12, 13). Sixth, no comparison-level CINeMA or GRADE certainty rating was conducted; therefore, the certainty of the antidepressant estimates remains limited (29, 30).

## Conclusion

In the source-verified antidepressant network, estimates for contemporary antidepressants (the SSRI fluoxetine, the SNRI levomilnacipran, and vilazodone, an SSRI and 5-HT1A receptor partial agonist) did not reach statistical significance versus pill placebo, although their confidence intervals remained compatible with small benefit as well as no effect. The only statistically significant estimate was derived from a small historical desipramine trial with important risk-of-bias and data-structure limitations. Current adolescent-specific arm-level evidence is too sparse and methodologically limited to support a stable comparative efficacy hierarchy among antidepressants. Future trials should use developmentally appropriate samples, prospectively specified outcomes, transparent arm-level reporting, and complete ascertainment and reporting of adverse events and suicidality.

## Data Availability

The original contributions presented in the study are included in the article/**Supplementary Material**. Further inquiries can be directed to the corresponding author.
