# Correction: Comparative efficacy of antidepressants for acute adolescent major depressive disorder: a source-verified systematic review and network meta-analysis

**DOI:** 10.3389/fpsyt.2026.2010890

**Published:** 2026-10-05

**Authors:** Chan Gong, Wengao Zeng, Xiangzhi Xiao, Qing Liu, Yaoyue Luo

**Affiliations:** 1 School of Nursing, Hunan University of Chinese Medicine, Changsha, Hunan, China; 2 Department of Internal Medicine, The Second Hospital of Changsha (West Branch of Changsha Hospital for Maternal and Child Health Care), Changsha, Hunan, China

**Keywords:** adolescent major depressive disorder, antidepressants, evidence robustness, network meta-analysis, reporting integrity, source verification

The funding from the Interdisciplinary Direction Cultivation Project of Hunan University of Chinese Medicine (Grant No. 2025JC0103) was erroneously omitted from the **Funding** statement.

In the published article, the **Funding** statement incorrectly read:

“The author(s) declared that financial support was not received for this work and/or its publication.”

The corrected **Funding** statement reads:

“The authors declare that financial support was received for this work and/or its publication. This work was supported by the Interdisciplinary Direction Cultivation Project of Hunan University of Chinese Medicine (Grant No. 2025JC0103).”

The original version of this article has been updated.

