# Supplementary material for: Comparative efficacy of antidepressants for acute adolescent major depressive disorder: a source-verified systematic review and network meta-analysis
Source: Front Psychiatry. 2026 Aug 28;17:1881200. doi: 10.3389/fpsyt.2026.1881200 (PMC13562017; doi:10.3389/fpsyt.2026.1881200)
Supplement: Supplementary file 1 [file SupplementaryFile1.docx]

**Supplementary Tables S1–S7 and Figures S1–S6**

**Supplementary Table S1. Source-document and trial/report classification in the strict re-adjudication corpus.**

| **File ID** | **Trial/report** | **Population and diagnosis** | **Data available** | **Analysis category** | **Final decision or reason** |
| --- | --- | --- | --- | --- | --- |
| P030 | von Knorring/citalopram PDF mismatch | Excluded from primary analysis: available PDF is a depression-screening article rather than the original randomized citalopram trial report. | No eligible primary arm-level endpoint mean (SD) retained | Excluded from quantitative synthesis | Excluded from quantitative synthesis because the available source was not the original randomized citalopram trial report, and eligible RCT treatment data could not be verified. |
| P001 | Hogberg 2018 | Not eligible for primary quantitative synthesis; DSM/ICD primary MDD diagnosis could not be verified for primary analysis | No eligible primary arm-level endpoint mean (SD) retained | Excluded from quantitative synthesis | Excluded from quantitative synthesis because DSM/ICD primary MDD eligibility and source-located arm-level data could not be verified from available sources. |
| P004 | Ahn 2011 | Excluded from primary analysis: drug evaluation/review, not an original randomized trial report. | No eligible primary arm-level endpoint mean (SD) retained | Excluded from quantitative synthesis | Excluded from quantitative synthesis. Drug evaluation/review, not an original RCT; retained for background and cross-checking of escitalopram evidence only. |
| P005 | Amr 2013 | Not eligible for primary quantitative synthesis; DSM/ICD MDD confirmed | No eligible primary arm-level endpoint mean (SD) retained | Excluded from quantitative synthesis | Excluded from quantitative synthesis. 7-14 sample and six-month endpoint; no eligible acute adolescent-only endpoint |
| P008 | Atkinson 2018 | Excluded from primary analysis: mixed child-adolescent sample; DSM-IV-TR MDD confirmed, but adolescent-only arm-level endpoint mean/SD was not retained for primary analysis. | No eligible primary arm-level endpoint mean (SD) retained | Excluded from quantitative synthesis | Excluded from quantitative synthesis. Mixed child-adolescent sample (7-17 years); no adolescent-only endpoint mean/SD retained for the primary analysis. |
| P010 | Clarke 2016 | Adolescent-only or trial-defined adolescent sample; DSM/ICD MDD confirmed | Disconnected direct evidence, CDRS-R, week 12 | Disconnected direct evidence and CDRS-R-only MD sensitivity | Not connected to the pill-placebo primary network; summarized separately. Adolescents 12-18 with major depression; not a duplicate. |
| P011 | Davey 2019 | Mixed adolescent-young adult MDD sample; participants aged 15-25 years with moderate-to-severe MDD based on SCID-IV. | No eligible primary arm-level endpoint mean (SD) retained | Narrative evidence only | Not included in the quantitative primary analysis. Mixed adolescent-young adult sample without source-located <=18 arm-level endpoint data. |
| P012 | Durgam 2018 | Adolescent-only or trial-defined adolescent sample; DSM/ICD MDD confirmed | Arm-level endpoint mean (SD), endpoint scale source-located, acute endpoint | Primary SMD and CDRS-R-only MD sensitivity | Included in the primary/sensitivity layer. Source verified week-8 endpoint mean (SD). |
| P013 | Kosik-Gonzalez 2026 | Adolescent-only or trial-defined adolescent sample; DSM/ICD MDD confirmed | No eligible primary arm-level endpoint mean (SD) retained | Special-population narrative evidence only | Narrative or special-population evidence only; not included in the primary analysis because the imminent-suicidal-risk add-on design was not commensurate with the pill-placebo primary network. |
| P016 | Emslie 2009 | Adolescent-only or trial-defined adolescent sample; DSM/ICD MDD confirmed | Adjusted/model-based contrast or LSM/SE data | Model-based sensitivity evidence | Included in model-based sensitivity only. Source verified; model-based only. |
| P019 | Fristad 2016 | Mixed child/adolescent depressive-disorder sample; DSM-IV-TR MDD, dysthymic disorder, or depressive disorder NOS. | No eligible primary arm-level endpoint mean (SD) retained | Excluded from quantitative synthesis | Excluded from quantitative synthesis. Same parent RCT as Young 2016; mixed 7-14-year-old depressive-disorder sample rather than adolescent-only primary MDD evidence. |
| P020 | Heiligenstein 2006 | Pediatric MDD nonresponders after 9-week acute fluoxetine treatment; DSM-IV MDD confirmed; special randomized reassignment/dose-escalation phase. | No eligible primary arm-level endpoint mean (SD) retained | Excluded from quantitative synthesis | Excluded from quantitative synthesis. Randomized reassignment after incomplete response to 9-week fluoxetine; special nonresponder/dose-escalation phase rather than an independent acute placebo-controlled primary-analysis trial. |
| P021 | Straub 2013 | Depressed adolescent inpatients with a major depressive episode/MDD; no qualifying randomized comparator identified. | No eligible primary arm-level endpoint mean (SD) retained | Excluded from quantitative synthesis | Excluded from quantitative synthesis. Nine inpatient adolescents; feasibility/pre-post style report, not a qualifying RCT. |
| P022 | Jensen-Doss 2018 / COMET | Excluded from primary analysis: protocol/design paper for transdiagnostic adolescent emotional-disorder treatment, not a completed randomized results report. | No eligible primary arm-level endpoint mean (SD) retained | Excluded from quantitative synthesis | Excluded from quantitative synthesis. Protocol/design paper, no extractable trial results. |
| P023 | Kobak 2015 | Not eligible for primary quantitative synthesis: DSM-5 mood/depressive disorders were included, but the sample was not primary MDD-only and the design was a clinician-level pilot implementation study. | No eligible primary arm-level endpoint mean (SD) retained | Excluded from quantitative synthesis | Excluded from quantitative synthesis. Clinician-level pilot implementation design and mixed depressive-disorder sample; not suitable as an independent arm-level MDD NMA RCT. |
| P024 | Kye 1996 | Adolescent-only or trial-defined adolescent sample; DSM/ICD MDD confirmed | Arm-level endpoint mean (SD), endpoint scale source-located, acute endpoint | Primary SMD | Included in the primary/sensitivity layer. Source verified ITT endpoint mean (SD); data-quality flag retained. |
| P025 | Miller 2018 | Not eligible for primary quantitative synthesis; DMDD/severe mood dysregulation sample, not a primary MDD trial. | No eligible primary arm-level endpoint mean (SD) retained | Excluded from quantitative synthesis | Excluded from quantitative synthesis. DMDD/severe mood dysregulation, not primary MDD trial. |
| P027 | Rice 2014 | Protocol for young people with MDD; mixed adolescent-young adult age range with planned DSM-IV assessment. | No eligible primary arm-level endpoint mean (SD) retained | Excluded from quantitative synthesis | Excluded from quantitative synthesis. Protocol paper for omega-3 in 15-25-year-olds; no results, mixed young adult age. |
| P028 | Stasiak 2012/2014 | Not eligible for primary quantitative synthesis; adolescents with depressive symptoms/low mood screened by rating-scale thresholds rather than source-verified DSM/ICD primary MDD. | No eligible primary arm-level endpoint mean (SD) retained | Excluded from quantitative synthesis | Excluded from quantitative synthesis. Depressive symptoms/low mood sample; source-verified DSM/ICD primary MDD diagnosis was not available for the primary analysis. |
| P029 | Grudin 2022 | Adolescent-only or trial-defined adolescent sample; DSM/ICD MDD confirmed | Disconnected direct evidence, CDRS-R, week 8 | Disconnected direct evidence and CDRS-R-only MD sensitivity | Not connected to the pill-placebo primary network; summarized separately. I-BA feasibility RCT in adolescents aged 13-17 with mild-to-moderate major depression. |
| P018 | TADS 2004 | Adolescent-only or trial-defined adolescent sample; DSM/ICD MDD confirmed | Adjusted endpoint values; not raw arm-level endpoint mean (SD) | Adjusted endpoint sensitivity evidence; not primary | Not eligible for primary endpoint mean (SD) analysis. Data are model-adjusted predicted scores; keep separate from primary arm-level endpoint mean/SD. |
| P017 | Israel 2012 | Not eligible for primary quantitative synthesis; DSM/ICD MDD confirmed | No eligible primary arm-level endpoint mean (SD) retained | Excluded from quantitative synthesis | Excluded from quantitative synthesis. Pilot Norwegian ABFT vs TAU, n=20; likely separate from Waraan 2021 larger ABFT trial but participant overlap with related reports could not be verified from available sources. |
| P026 | Radecki 2024 LVM-MD-11 | Adolescent-only or trial-defined adolescent sample; DSM/ICD MDD confirmed | Arm-level endpoint mean (SD), CDRS-R, week 8 | Primary SMD and CDRS-R-only MD sensitivity; model-based sensitivity | Included in the primary/sensitivity layer. Source verified week-8 endpoint mean (SD); data-quality flag retained. |
| P026 | Radecki 2024 LVM-MD-14 | Excluded from primary analysis: mixed child-adolescent sample; DSM/ICD MDD confirmed, but separate adolescent-only arm-level endpoint mean/SD was not retained for primary analysis. | No eligible primary arm-level endpoint mean (SD) retained | Excluded from quantitative synthesis | Excluded from quantitative synthesis. Mixed 7-17 sample; no separate adolescent-only efficacy data retained. |
| P031 | Findling 2022 | Adolescent-only or trial-defined adolescent sample; DSM/ICD MDD confirmed | Reported MMRM treatment differences or adjusted/model-based contrast data; no raw arm-level endpoint mean (SD) retained for primary analysis | Model-based sensitivity evidence | Included in model-based sensitivity only. Source verified; reported MMRM/LS mean differences were used where available; not combined with primary endpoint mean/SD data. |
| P032 | Wagner 2003 | Excluded from primary analysis: pooled pediatric 6-17 randomized trials with DSM-IV MDD; adolescent-only arm-level endpoint mean/SD was not retained for primary analysis. | No eligible primary arm-level endpoint mean (SD) retained | Excluded from quantitative synthesis | Excluded from quantitative synthesis. Pooled pediatric 6-17 trials; no adolescent-only randomized arm-level endpoint data retained. |
| P033 | Wagner 2006 | Excluded from primary analysis: pediatric 6-17 randomized trial with MDD; adolescent-only findings were post hoc and not retained as randomized arm-level main data for primary analysis. | No eligible primary arm-level endpoint mean (SD) retained | Excluded from quantitative synthesis | Excluded from quantitative synthesis. Pediatric 6-17 trial; adolescent-only values are post hoc and not randomized arm-level main data. |
| P034 | Waraan 2021 | Not eligible for primary quantitative synthesis; DSM/ICD MDD confirmed, but the randomized treatment endpoint was 16 weeks and no eligible <=12-week arm-specific endpoint mean/SD was retained. | No eligible primary arm-level endpoint mean (SD) retained | Narrative evidence only | Narrative only; not included in quantitative synthesis because the randomized treatment endpoint was 16 weeks and no eligible <=12-week arm-specific endpoint mean/SD was retained. |
| P035 | Weihs 2018 | Excluded from primary analysis: mixed child-adolescent sample; DSM-IV-TR MDD confirmed, but adolescent-only arm-level endpoint mean/SD was not retained for primary analysis. | No eligible primary arm-level endpoint mean (SD) retained | Excluded from quantitative synthesis | Excluded from quantitative synthesis. Mixed child-adolescent trial (7-17 years); no adolescent-only endpoint mean/SD retained. |
| P036 | Wright 2017 | Not eligible for primary quantitative synthesis; adolescents with low mood/depression screened by MFQ threshold rather than source-verified DSM/ICD primary MDD. | No eligible primary arm-level endpoint mean (SD) retained | Excluded from quantitative synthesis | Excluded from quantitative synthesis. Low mood/depression sample; source-verified DSM/ICD primary MDD diagnosis and eligible primary-analysis outcomes were not available. |
| P037 | Young 2016 | Mixed child/adolescent DSM-IV-TR depressive-disorder sample; secondary behavioral-outcome report from the Omega-3 + PEP pilot RCT. | No eligible primary arm-level endpoint mean (SD) retained | Excluded from quantitative synthesis | Excluded from quantitative synthesis. Same Omega-3 + PEP pilot RCT as Fristad 2016; secondary behavioral outcomes, not a separate parent trial. |
| P002 | Simeon 1990 | Not eligible for primary quantitative synthesis; DSM/ICD MDD confirmed | No eligible primary arm-level endpoint mean (SD) retained | Narrative evidence only | Not included in the quantitative primary analysis. Eligible trial but no source-located arm-level endpoint mean/SD or contrast SE in available article text. |
| P003 | Klein 1998 | Adolescent-only or trial-defined adolescent sample; DSM/ICD MDD confirmed | HAM-D week-6 arm-level endpoint mean (SD) used for primary SMD; BDI available as secondary patient-rated outcome but not used in the primary network | Primary SMD and binary direct sensitivity | Included in the primary/sensitivity layer. Source verified completer endpoint; reported pooled SD; data-quality flag retained. |
| P006 | Rohde 2004 | Adolescent-only or trial-defined adolescent sample; DSM/ICD MDD confirmed | Binary response/remission data | Binary direct sensitivity; disconnected direct evidence | Not connected to the pill-placebo primary network; summarized separately. Adolescents 13-17 with MDD and conduct disorder randomized to CWD-A vs life skills/tutoring. |
| P007 | Berard 2006 | Not included in the primary analysis because source-located adolescent-only arm-level endpoint mean/SD data were unavailable; retained only as age-boundary, model-based, or binary sensitivity evidence where applicable; DSM/ICD MDD confirmed | Adjusted/model-based contrast or LSM/SE data | Model-based or binary sensitivity evidence | Included in model-based sensitivity only. Source verified; retained only for model-based or binary sensitivity evidence where applicable. |
| P009 | Diamond 2002 | Adolescent-only or trial-defined adolescent sample; DSM/ICD MDD confirmed | No eligible primary arm-level endpoint mean (SD) retained | Disconnected direct or narrative evidence | Not connected to the pill-placebo primary network; summarized separately. ABFT treatment development study; not duplicate with Israel/Waraan ABFT Norway trials. |
| P014 | Mufson 1999 | Adolescent-only or trial-defined adolescent sample; DSM/ICD MDD confirmed | No eligible primary arm-level endpoint mean (SD) retained | Disconnected direct or narrative evidence | Not connected to the pill-placebo primary network; summarized separately. 12-week IPT-A RCT in 12-18 DSM-III-R MDD adolescents. |
| P015 | Keller 2001 | Adolescent-only or trial-defined adolescent sample; DSM/ICD MDD confirmed | Adjusted/model-based contrast or LSM/SE data | Model-based sensitivity evidence | Included in model-based sensitivity only. Source verified; model-based only; covariance unavailable. |

Note. The counting unit is a trial/report classification entry, not an eligible parent trial. Thirty-seven source documents (P001-P037) yielded 38 entries because P026 (Radecki 2024) reported two trials (LVM-MD-11 and LVM-MD-14). The table includes primary, sensitivity, disconnected, narrative, and excluded records: 4 parent-trial entries contributed to the primary SMD network, 14 entries contributed only to other evidence layers or narrative synthesis, and 20 entries were excluded from quantitative synthesis. The legacy broad analysis contained 51 study identifiers and is not presented as a 51-to-38 report-to-parent-trial conversion.

**Supplementary Table S2. Primary arm-level source verification table.**

| **Trial** | **Treatment arm** | **n** | **Scale and endpoint** | **Endpoint mean (SD)** | **Source** | **Data-quality note** |
| --- | --- | --- | --- | --- | --- | --- |
| Klein 1998 | Desipramine | 18 | HAM-D, week 6 | 10.83 (2.10) | pp. 10-11, Table 6 | Completer endpoint; reported common pooled SD |
| Klein 1998 | Pill placebo | 18 | HAM-D, week 6 | 14.61 (2.10) | pp. 10-11, Table 6 | Completer endpoint; reported common pooled SD |
| Kye 1996 | Amitriptyline | 18 | HAM-D, week 8 | 8.00 (4.90) | pp. 3-4, Results; Table 2 | Reported ITT protocol-end endpoint; manual ITT/completer flag retained |
| Kye 1996 | Pill placebo | 13 | HAM-D, week 8 | 8.80 (4.50) | pp. 3-4, Results; Table 2 | Reported ITT protocol-end endpoint; manual ITT/completer flag retained |
| Durgam 2018 | Vilazodone 15 mg | 174 | CDRS-R, week 8 | 33.80 (12.00) | p. 7, Table 2 | ITT observed week-8 endpoint; not an LSM change |
| Durgam 2018 | Vilazodone 30 mg | 180 | CDRS-R, week 8 | 32.50 (11.50) | p. 7, Table 2 | ITT observed week-8 endpoint; not an LSM change |
| Durgam 2018 | Pill placebo | 170 | CDRS-R, week 8 | 34.00 (12.90) | p. 7, Table 2 | ITT observed week-8 endpoint; not an LSM change |
| Radecki 2024 LVM-MD-11 | Levomilnacipran 40 mg | 134 | CDRS-R, week 8 | 38.10 (12.60) | p. 6, Table 2 | Observed week-8 endpoint; LSM/SE data kept separate |
| Radecki 2024 LVM-MD-11 | Levomilnacipran 80 mg | 138 | CDRS-R, week 8 | 37.60 (12.70) | p. 6, Table 2 | Observed week-8 endpoint; LSM/SE data kept separate |
| Radecki 2024 LVM-MD-11 | Fluoxetine | 134 | CDRS-R, week 8 | 37.50 (14.20) | p. 6, Table 2 | Observed week-8 endpoint; LSM/SE data kept separate |
| Radecki 2024 LVM-MD-11 | Pill placebo | 140 | CDRS-R, week 8 | 39.60 (15.30) | p. 6, Table 2 | Observed week-8 endpoint; LSM/SE data kept separate |

Note. This table lists each parent trial, treatment arm, n, endpoint mean, SD, scale, endpoint week, source page/table, ITT/completer/protocol-end status, data-quality flag, and primary SMD eligibility decision. Values are endpoint mean (SD); lower HAM-D and CDRS-R scores indicate fewer depressive symptoms. p., page; pp., pages; HAM-D, Hamilton Depression Rating Scale; CDRS-R, Children's Depression Rating Scale-Revised; ITT, intention-to-treat; LSM, least-squares mean; SE, standard error. Demographic or safety denominators may differ from endpoint or ITT denominators.

**Supplementary Table S3. Primary SMD estimates versus pill placebo and influence analyses excluding historical trials evaluating tricyclic antidepressants.**

| **Analysis** | **Treatment** | **Reference** | **Hedges' g SMD (95% CI)** | **Statistically significant** | **Contributing parent trials** | **Treatment arms** |
| --- | --- | --- | --- | --- | --- | --- |
| Primary analysis | Desipramine | Pill placebo | -1.80 [-2.57, -1.03] | Yes | 4 | 11 |
| Primary analysis | Amitriptyline | Pill placebo | -0.17 [-0.88, 0.55] | No | 4 | 11 |
| Primary analysis | Fluoxetine | Pill placebo | -0.15 [-0.39, 0.08] | No | 4 | 11 |
| Primary analysis | Levomilnacipran 80 mg | Pill placebo | -0.15 [-0.38, 0.09] | No | 4 | 11 |
| Primary analysis | Vilazodone 30 mg | Pill placebo | -0.12 [-0.33, 0.09] | No | 4 | 11 |
| Primary analysis | Levomilnacipran 40 mg | Pill placebo | -0.11 [-0.35, 0.13] | No | 4 | 11 |
| Primary analysis | Vilazodone 15 mg | Pill placebo | -0.02 [-0.23, 0.19] | No | 4 | 11 |
| Influence analysis excluding Klein 1998 | Amitriptyline | Pill placebo | -0.17 [-0.88, 0.55] | No | 3 | 9 |
| Influence analysis excluding Klein 1998 | Fluoxetine | Pill placebo | -0.15 [-0.39, 0.08] | No | 3 | 9 |
| Influence analysis excluding Klein 1998 | Levomilnacipran 80 mg | Pill placebo | -0.15 [-0.38, 0.09] | No | 3 | 9 |
| Influence analysis excluding Klein 1998 | Vilazodone 30 mg | Pill placebo | -0.12 [-0.33, 0.09] | No | 3 | 9 |
| Influence analysis excluding Klein 1998 | Levomilnacipran 40 mg | Pill placebo | -0.11 [-0.35, 0.13] | No | 3 | 9 |
| Influence analysis excluding Klein 1998 | Vilazodone 15 mg | Pill placebo | -0.02 [-0.23, 0.19] | No | 3 | 9 |
| Influence analysis excluding Klein 1998 and Kye 1996 | Fluoxetine | Pill placebo | -0.15 [-0.39, 0.08] | No | 2 | 7 |
| Influence analysis excluding Klein 1998 and Kye 1996 | Levomilnacipran 80 mg | Pill placebo | -0.15 [-0.38, 0.09] | No | 2 | 7 |
| Influence analysis excluding Klein 1998 and Kye 1996 | Vilazodone 30 mg | Pill placebo | -0.12 [-0.33, 0.09] | No | 2 | 7 |
| Influence analysis excluding Klein 1998 and Kye 1996 | Levomilnacipran 40 mg | Pill placebo | -0.11 [-0.35, 0.13] | No | 2 | 7 |
| Influence analysis excluding Klein 1998 and Kye 1996 | Vilazodone 15 mg | Pill placebo | -0.02 [-0.23, 0.19] | No | 2 | 7 |

Note. Values are Hedges' g SMDs with 95% CIs versus pill placebo. Negative values favor the active treatment. The influence analyses exclude historical trials evaluating TCAs as indicated in the analysis column. CI, confidence interval; TCA, tricyclic antidepressant.

**Supplementary Table S4. Model-based contrast sensitivity evidence, including contrast source type and data-quality flag.**

| **Trial** | **Treatment** | **Reference** | **Effect estimate or reported summary** | **Scale/week** | **Source type** | **Data-use note** |
| --- | --- | --- | --- | --- | --- | --- |
| Findling 2022 | Fluoxetine | Pill placebo | -3.70 (SE 1.5; p=0.015) | CDRS-R, week 8 | Reported MMRM treatment difference versus placebo; FAS | Source verified. Reported MMRM/LS mean difference used directly; model-based sensitivity only; not combined with primary endpoint mean/SD data. |
| Emslie 2009 | Escitalopram | Pill placebo | -3.356 [-6.226, -0.486] | CDRS-R, week 8 | Reported active-vs-placebo LSMD with 95% CI | Source verified. Reported LSMD used directly; model-based/adjusted endpoint contrast only; not combined with primary endpoint mean/SD data. |
| Keller 2001 / Study 329 | Paroxetine | Pill placebo | -1.64 [-3.91, 0.63] | HAM-D, week 8 | Derived from reported adjusted endpoint mean (SE); covariance unavailable | Source verified; model-based only; covariance unavailable for derived contrast. |
| Radecki 2024 LVM-MD-11 | Fluoxetine | Pill placebo | -1.47 [-4.5, 1.6] | CDRS-R, week 8 | Reported LSM change versus placebo with 95% CI; ITT | Source verified. Reported LSM contrast used in model-based layer only; kept separate from observed endpoint mean/SD primary layer. |
| Findling 2022 | Vortioxetine 20 mg | Pill placebo | -0.70 (SE 1.5; p=0.637) | CDRS-R, week 8 | Reported MMRM treatment difference versus placebo; FAS | Source verified. Reported MMRM/LS mean difference used directly; model-based sensitivity only; not combined with primary endpoint mean/SD data. |
| Keller 2001 / Study 329 | Imipramine | Pill placebo | -0.68 [-2.95, 1.59] | HAM-D, week 8 | Derived from reported adjusted endpoint mean (SE); covariance unavailable | Source verified; model-based only; covariance unavailable for derived contrast. |
| Radecki 2024 LVM-MD-11 | Levomilnacipran 40 mg | Pill placebo | -0.38 [-3.4, 2.6] | CDRS-R, week 8 | Reported LSM change versus placebo with 95% CI; ITT | Source verified. Reported LSM contrast used in model-based layer only; kept separate from observed endpoint mean/SD primary layer. |
| Radecki 2024 LVM-MD-11 | Levomilnacipran 80 mg | Pill placebo | 0.26 [-2.8, 3.3] | CDRS-R, week 8 | Reported LSM change versus placebo with 95% CI; ITT | Source verified. Reported LSM contrast used in model-based layer only; kept separate from observed endpoint mean/SD primary layer. |
| Findling 2022 | Vortioxetine 10 mg | Pill placebo | 1.10 (SE 1.6; p=0.470) | CDRS-R, week 8 | Reported MMRM treatment difference versus placebo; FAS | Source verified. Reported MMRM/LS mean difference used directly; model-based sensitivity only; not combined with primary endpoint mean/SD data. |

Note. Model-based least-squares mean differences, MMRM treatment differences, adjusted endpoint estimates, and derived contrasts are shown on their source scales and were not combined with the primary endpoint mean/SD SMD network. Reported active-versus-placebo estimates were used when available. Derived contrasts for which covariance information was unavailable are identified in the source-type and data-quality fields.

**Supplementary Table S5. Binary direct sensitivity evidence for response/remission outcomes.**

| **Trial** | **Outcome** | **Definition** | **Week** | **Treatment vs reference** | **Events/N** | **OR (95% CI)** |
| --- | --- | --- | --- | --- | --- | --- |
| Klein 1998 desipramine placebo | Remission | No longer meeting MDD criteria | 6 | Desipramine vs Pill placebo | 13/18 vs 9/18 | 2.60 [0.65, 10.38] |
| Klein 1998 desipramine placebo | Response | Psychiatrist CGI rating 1-3 | 6 | Desipramine vs Pill placebo | 12/18 vs 9/18 | 2.00 [0.52, 7.69] |
| Rohde 2004 CWD-A versus life skills | Remission | Recovery from DSM-IV MDD | 8 | CBT vs Active control | 17/44 vs 9/47 | 2.66 [1.03, 6.85] |

Note. Binary outcomes are reported as direct evidence because outcome definitions and network components differed across studies. OR > 1 favors the first-listed treatment for response or remission outcomes. OR, odds ratio; CI, confidence interval.

**Supplementary Table S6. Trial-level Cochrane RoB 2 and data-structure assessment.**

| **Trial/report** | **Analysis category** | **D1: Randomization process** | **D2: Deviations from intended interventions** | **D3: Missing outcome data** | **D4: Outcome measurement** | **D5: Selection of reported result** | **Overall RoB 2 judgment** | **Data-structure note** | **Data-use note** |
| --- | --- | --- | --- | --- | --- | --- | --- | --- | --- |
| Klein 1998 | Primary SMD; influence analysis excluding historical trials evaluating TCAs | Some concerns: randomization/allocation details were incompletely reported in the historical trial report. | Some concerns: double-blind design, but limited reporting of deviations from intended interventions. | High risk: primary endpoint values used completer data in a small sample. | Some concerns: clinician-rated HAM-D outcome; blinding reported but historical reporting details limited. | Some concerns: historical reporting standards and limited protocol availability. | High risk | HAM-D endpoint mean/SD; reported pooled/common SD; source verified with explicit completer and pooled-SD flags. | Included in the full primary SMD network; interpretation supported by influence analysis excluding Klein 1998. |
| Kye 1996 | Primary SMD; influence analysis excluding historical trials evaluating TCAs | Some concerns: randomization details were not fully reported by current reporting standards. | Some concerns: randomized placebo-controlled design, but reporting of deviations was limited. | Some concerns: small sample; ITT/protocol-end distinction retained as a data-quality flag. | Some concerns: HAM-D outcome measurement; blinding reported but historical reporting details limited. | Some concerns: limited protocol-level reporting and historical reporting standards. | Some concerns | HAM-D week-8 endpoint mean/SD; source verified; ITT/protocol-end endpoint flag retained. | Included in the full primary SMD network; excluded with Klein 1998 in the influence analysis excluding historical trials evaluating TCAs. |
| Durgam 2018 | Primary SMD; CDRS-R-only MD connected sensitivity | Low risk: randomized double-blind placebo-controlled trial. | Low risk: blinded pharmacotherapy design with protocolized treatment. | Some concerns: ITT endpoint data were used, but individual-level missing-data assumptions could not be reanalyzed. | Low risk: CDRS-R was a standard depressive-symptom outcome and blinding was maintained. | Some concerns: industry-sponsored trial; no RIAT reanalysis identified. | Some concerns | Observed week-8 CDRS-R endpoint mean/SD; source verified; not an LSM change. Safety/demographic denominators may differ from endpoint/ITT denominators. | Included in the primary SMD and CDRS-R-only connected MD sensitivity analyses. |
| Radecki 2024 LVM-MD-11 | Primary SMD; CDRS-R-only MD connected sensitivity; model-based sensitivity | Low risk: randomized double-blind placebo-controlled trial with fluoxetine reference arm. | Low risk: blinded pharmacotherapy design with protocolized treatment. | Some concerns: ITT endpoint data were used; individual-level missing-data assumptions could not be reanalyzed. | Low risk: CDRS-R was a standard depressive-symptom outcome and blinding was maintained. | Some concerns: industry-sponsored trial; no RIAT reanalysis identified. | Some concerns | Observed week-8 CDRS-R endpoint mean/SD used for primary SMD; LSM/SE and reported LSM differences kept in a separate model-based layer. Safety/demographic denominators may differ from endpoint/ITT denominators. | Included in primary and sensitivity layers with explicit separation of observed endpoint and model-based estimates. |
| Findling 2022 | Model-based active-versus-placebo direct sensitivity | Low risk: randomized placebo-controlled trial with fluoxetine reference arm. | Low risk: blinded pharmacotherapy design with protocolized treatment. | Some concerns: model-based estimates were used; individual-level missing-data assumptions could not be reanalyzed. | Low risk: CDRS-R was a standard depressive-symptom outcome and blinding was maintained. | Some concerns: industry-sponsored trial; model-based sensitivity evidence was based on reported MMRM/LS mean differences rather than raw arm-level endpoint mean/SD data. | Some concerns | Reported MMRM/LS mean differences versus placebo were available for sensitivity interpretation; not primary raw endpoint mean/SD data. | Summarized as model-based sensitivity evidence; not combined with primary endpoint mean/SD data. |
| Emslie 2009 | Model-based active-versus-placebo direct sensitivity | Low risk: randomized placebo-controlled escitalopram adolescent trial. | Low risk: blinded pharmacotherapy design with protocolized treatment. | Some concerns: model-based or adjusted estimates were used; individual-level missing-data assumptions could not be reanalyzed. | Low risk: CDRS-R was a standard depressive-symptom outcome and blinding was maintained. | Some concerns: industry-sponsored trial; raw arm-level endpoint mean/SD not used in the primary network. | Some concerns | Model-based or adjusted estimates available for sensitivity interpretation; not primary raw endpoint mean/SD data. | Summarized as model-based sensitivity evidence only. |
| Keller 2001 / Study 329 | Model-based and interpretive evidence | Some concerns: randomized placebo-controlled trial, but trial conduct and reporting were subject to post-publication scrutiny. | Some concerns: deviations cannot be fully evaluated from the published report alone. | Some concerns: missing-data handling and analysis population were part of later reporting concerns. | Some concerns: depressive-symptom outcome measurement was standard, but harms and outcomes were affected by reporting concerns. | High risk: major post-publication RIAT reanalysis and selective-reporting concerns. | High risk | Adjusted/model-based endpoint estimates; not included as primary raw arm-level endpoint mean/SD evidence. | Summarized in model-based or interpretive evidence layers; not included in the primary SMD network. |
| TADS / March 2004 | Adjusted endpoint not primary; interpretive evidence | Some concerns: large randomized adolescent depression trial, but design and blinding differed across treatment arms. | Some concerns: psychotherapy and pharmacotherapy components create different possibilities for deviations from intended interventions. | Some concerns: adjusted/model-derived values were available; raw arm-level endpoint mean/SD was not used for the primary network. | Some concerns: outcome assessment was structured, but treatment masking differed across arms. | Some concerns: subsequent RIAT reanalysis and unblinding/reporting concerns support cautious interpretation. | Some concerns | Source-located values available for this review were adjusted or model-derived rather than raw arm-level endpoint mean/SD data. | Discussed as contextual evidence; not included in the primary endpoint mean/SD SMD network. |
| Clarke 2016 | Disconnected direct evidence; CDRS-R-only MD disconnected sensitivity | Some concerns: randomization was reported, but allocation details were not fully verifiable from extracted information. | Some concerns: psychotherapy/TAU comparison could not be fully blinded. | Some concerns: small trial and missing-data assumptions could not be reanalyzed. | Some concerns: depressive-symptom outcome measurement was standard, but blinding of participants/therapists was not possible. | Some concerns: limited evidence layer and no formal comparison-level certainty assessment. | Some concerns | Endpoint data summarized in disconnected direct-evidence layer; not connected to pill-placebo primary network. | Reported separately as direct evidence; not included in the primary pharmacotherapy NMA. |
| Grudin 2022 | Disconnected direct evidence; CDRS-R-only MD disconnected sensitivity | Some concerns: feasibility RCT with limited reporting details for allocation procedures. | Some concerns: behavioral activation/TAU comparison could not be fully blinded. | Some concerns: small trial and missing-data assumptions could not be reanalyzed. | Some concerns: depressive-symptom outcome measurement was standard, but blinding was limited by intervention type. | Some concerns: feasibility design and limited reporting reduce certainty of the direct estimate. | Some concerns | Endpoint data summarized in disconnected direct-evidence layer; not connected to pill-placebo primary network. | Reported separately as direct evidence; not included in the primary pharmacotherapy NMA. |
| Rohde 2004 | Binary direct sensitivity; disconnected direct evidence | Some concerns: randomized active-control psychotherapy trial, but allocation details were not fully verifiable from extracted information. | Some concerns: psychotherapy comparison could not be fully blinded. | Some concerns: binary outcomes and missing-data assumptions could not be reanalyzed from aggregate data. | Some concerns: diagnostic recovery/remission outcomes were clinically relevant but assessment masking was unclear from available extracted information. | Some concerns: disconnected single-study evidence layer and limited protocol-level reporting. | Some concerns | Binary response/remission data; disconnected from pill-placebo primary network. | Reported separately as binary direct evidence; not included in the primary pharmacotherapy NMA. |

Note. This table reports domain-level and overall RoB 2 judgments for trials represented in the primary or supplementary analyses, alongside endpoint-data structure, missing-data concerns, and post-publication reporting concerns relevant to interpretation. It supports risk-of-bias interpretation but does not constitute a comparison-level CINeMA or GRADE certainty rating. RoB 2, Cochrane Risk of Bias 2 tool for randomized trials; TCA, tricyclic antidepressant; SMD, standardized mean difference; MD, mean difference; CDRS-R, Children's Depression Rating Scale-Revised; HAM-D, Hamilton Depression Rating Scale; LSM, least-squares mean; SE, standard error; RIAT, restoring invisible and abandoned trials.

**Supplementary Table S7. Source-verified study characteristics of source reports considered in the systematic review.**

Note. This table summarizes report type, population and diagnosis, age and female participants, treatment arms and sample sizes, endpoint timing, outcome measures, analysis category, and source-verification notes, including denominator distinctions where randomized, safety, treated/demographic, FAS, and endpoint/ITT denominators differ. NR indicates that the field was not reliably source-located.

| **No.** | **Source report / type** | **Population and diagnosis** | **Age / female participants** | **Treatment arms and sample size** | **Duration / endpoint** | **Outcome measure(s)** | **Analysis category / final use** | **Source-verification note** |
| --- | --- | --- | --- | --- | --- | --- | --- | --- |
| 1 | von Knorring / citalopram PDF mismatch; non-trial/mismatched source | Depression-screening article, not the original randomized citalopram trial report; DSM-IV depression-screening sample reported. | 13-20 years; 104 girls/127 screened in source article; not a randomized treatment sample. | No eligible randomized treatment arms in available source. | NA | BDI / DSM-IV depression screening reported in available source. | Excluded from quantitative synthesis. | The available source was not treated as an original citalopram RCT; excluded because the available source did not match the original citalopram RCT. |
| 2 | Hogberg 2018; randomized psychotherapy study | Depressed/suicidal adolescents in a clinical setting; DSM/ICD primary MDD eligibility not verified for primary analysis. | Mean age 14.2 years (MR-CBT) and 15.2 years (TAU); 19 female, 7 male, 1 undetermined gender in treatment sample. | MR-CBT n=15 vs TAU n=12. | Median MR-CBT treatment period 8 months; not an acute <=12-week endpoint. | SMFQ, WHO-5, C-SSRS. | Excluded from quantitative synthesis. | Not retained for primary NMA because DSM/ICD MDD eligibility and compatible arm-level endpoint mean/SD data were not verified. |
| 3 | Ahn 2011; drug evaluation/review | Review/expert evaluation of escitalopram in youth, not an original RCT. | NA | No original randomized arms. | NA | Narrative review of escitalopram/citalopram evidence. | Excluded from quantitative synthesis; background/cross-check only. | This report was not counted as an independent RCT. |
| 4 | Amr 2013; randomized double-blind placebo-controlled pilot study | Pediatric MDD; fluoxetine plus vitamin C adjunctive trial. | Eligibility: pediatric patients <18 years; completer/analyzed sample 7-14 years; mean age 10.3 vs 9.9 years; sex M/F 7/5 vs 8/4 among completers. | Fluoxetine + vitamin C initially assigned n=14 vs fluoxetine + placebo initially assigned n=13; completer/analyzed sample n=12 vs n=12. | 6 months; not acute <=12-week endpoint. | CDRS, CDI, CGI. | Excluded from quantitative synthesis. | PDF-verified. Eligibility was <18 years with DSM-IV-TR MDD, but the analyzed/completer sample was 7-14 years and the treatment endpoint was 6 months; therefore excluded from the adolescent-only acute quantitative synthesis. |
| 5 | Atkinson 2018; randomized double-blind placebo-controlled trial | Children/adolescents with DSM-IV-TR MDD; mixed 7-17 sample. | 7-17 years; children n=109, adolescents n=254; female 205/363 overall; adolescents female 159/254. | Placebo n=120; low-exposure desvenlafaxine n=122; high-exposure desvenlafaxine n=121. | 8 weeks. | CDRS-R, CGI-S, CGI-I, C-SSRS. | Excluded from quantitative synthesis. | Mixed child-adolescent trial; adolescent-only arm-level endpoint mean/SD not retained for primary analysis. |
| 6 | Clarke 2016; randomized trial | Adolescents with major depression who declined/discontinued antidepressant treatment. | 12-18 years; girls 145/212 (68.4%). | TAU + brief individual CBT n=106 vs TAU control n=106. | Assessed through 2 years; week-12 CDRS-R available as acute/disconnected evidence. | Major depression diagnostic recovery, CDRS-R, CES-D. | Disconnected direct evidence and CDRS-R-only MD sensitivity. | PDF-verified. Participants/baseline text reports 145/212 girls (68.4%). Not connected to pill-placebo primary pharmacotherapy network; summarized separately. |
| 7 | Davey 2019 / YoDA-C; randomized double-blind placebo-controlled multicentre trial | Youth with moderate-to-severe MDD; mixed adolescent-young adult sample. | 15-25 years; mean 19.6 years; <18 years n=49/153; female 90/153. | CBT + placebo n=77 vs CBT + fluoxetine n=76. | 12 weeks. | MADRS primary; other safety/suicidality measures. | Narrative evidence only. | Mixed age sample; no source-located <=18 arm-level endpoint data for primary analysis. |
| 8 | Durgam 2018; phase 3 randomized double-blind placebo-controlled trial | Adolescents with DSM-IV-TR MDD, CDRS-R >=40 and CGI-S >=4. | 12-17 years; female 103/171, 103/175, 107/180 in table by placebo/vilazodone dose groups. | Placebo n=174; vilazodone 15 mg/day n=175; vilazodone 30 mg/day n=180. | 8-week double-blind treatment; 10-week study including screening/down-taper. | CDRS-R primary, CGI-S secondary, C-SSRS. | Primary SMD and CDRS-R-only MD sensitivity. | Source-verified observed week-8 endpoint mean/SD retained; not model-based LSM change for primary SMD. Female counts are from the baseline/safety population; endpoint/ITT denominators used for primary endpoint extraction differ for some arms. |
| 9 | Kosik-Gonzalez 2026; randomized double-dummy psychoactive-controlled study | Adolescents with MDD at imminent suicide risk; special-population add-on design with comprehensive standard of care. | 12 to <18 years; female 114/146 in baseline table. | Esketamine 28/56/84 mg vs oral midazolam; randomized 1:1:1:2; total randomized 147. | 4 weeks; primary 24-hour post-first-dose endpoint. | CDRS-R, CGI-SS-R, safety/adverse events. | Special-population narrative evidence only. | Not commensurate with pill-placebo primary network due imminent-suicidal-risk add-on design and psychoactive comparator. |
| 10 | Emslie 2009; randomized placebo-controlled multisite trial | Adolescents with DSM-IV-defined MDD. | 12-17 years; mean age approximately 14.5-14.7 years; female 92/157 placebo and 92/155 escitalopram in the baseline/demographic table. Efficacy denominator differs for some outcomes. | Escitalopram 10-20 mg/day n=155 vs placebo n=157. | 8 weeks. | CDRS-R primary; adverse events/suicidality. | Model-based sensitivity evidence. | PDF-verified from baseline/demographic table. Sex counts are baseline/demographic denominators, not necessarily the endpoint efficacy denominator. Model-based/adjusted estimates retained separately from primary arm-level endpoint mean/SD layer. |
| 11 | Fristad 2016; pilot 2x2 randomized controlled trial | Youth with major depression, dysthymia, or depression NOS; mixed child/adolescent sample. | 7-14 years; 57% male (43% female). | Omega-3, PEP+placebo, omega-3+PEP, placebo; total N=72. | 12 weeks. | CDRS-R; remission; side effects. | Excluded from quantitative synthesis. | Same parent RCT as Young 2016; not adolescent-only DSM/ICD MDD primary quantitative evidence. |
| 12 | Heiligenstein 2006; randomized reassignment/dose-escalation study | Pediatric MDD nonresponders after 9-week acute fluoxetine treatment; special nonresponder phase. | 9-17 years in randomized reassignment phase; female by arm: fluoxetine 20 mg 6/15; fluoxetine 40-60 mg 5/14. | Fluoxetine 40-60 mg/day n=14 vs fluoxetine 20 mg/day n=15. | Additional 10 weeks after initial 9-week fluoxetine phase. | CDRS-R response/improvement. | Excluded from quantitative synthesis. | PDF-verified. Table 1 patient demographics reports female counts 6/15 and 5/14. Randomized reassignment after incomplete response; not an independent acute placebo-controlled primary-analysis trial. |
| 13 | Straub 2013; feasibility/pre-post report | Depressive adolescent inpatients; no qualifying randomized comparator identified. | Abstract reports mean age 16.33 years; participants section reports mean age 16.43 years and female 5/9. | MICHI group intervention; n=9. | Brief inpatient group programme; exact endpoint NR. | CDRS-R; suicidal ideation item; feasibility ratings. | Excluded from quantitative synthesis. | PDF-verified. Participants section reports nine depressed adolescent inpatients, mean age 16.43 years, five girls. Feasibility/pre-post style report; not a qualifying RCT for analysis. |
| 14 | Jensen-Doss 2018 / COMET; protocol/design paper | Protocol for transdiagnostic treatment and measurement feedback in adolescents with emotional disorders. | Adolescents; exact randomized sample not available because protocol. | Planned TAU, TAU+YOQ, UP-A+YOQ arms; no results. | Protocol only. | Planned emotional-disorder outcomes. | Excluded from quantitative synthesis. | Design/protocol paper with no extractable trial results. |
| 15 | Kobak 2015; pilot implementation/clinician-randomized study | Adolescents with DSM-5 mood/depressive disorders treated by randomized clinicians; mixed depressive-disorder sample, not primary MDD-only. | Patients aged 12-17; mean 15.4 years; female 43/65. Diagnoses included MDD, persistent depressive disorder, both, other specified depressive disorder, and unspecified depressive disorder. | Clinicians randomized to technology-enhanced CBT vs TAU; 18 clinicians, each treating up to four adolescents; 65 adolescents described. | 12 weeks. | QIDS-A-Pat, TASA, usability/knowledge outcomes. | Excluded from quantitative synthesis. | PDF-verified. Patient characteristics report 65 adolescents and two-thirds female (n=43). Clinician-level pilot implementation design and mixed depressive-disorder sample; not suitable as independent arm-level MDD NMA RCT. |
| 16 | Kye 1996; randomized double-blind placebo-controlled trial | Adolescents with Research Diagnostic Criteria MDD confirmed by K-SADS. | 12-17 years; female by arm: amitriptyline 5/18; placebo 4/13 (total 9/31). | Amitriptyline n=18 vs placebo n=13. | 8 weeks after 2-week placebo washout. | HAM-D, K-SADS nine-item scale, CGI. | Primary SMD. | PDF-verified. Baseline demographics paragraph reports male/female ratio 13/5 in the amitriptyline arm and 9/4 in the placebo arm. Source-verified ITT/protocol-end endpoint mean/SD retained with data-quality flag. |
| 17 | Miller 2018; pilot randomized psychosocial trial | Youth with DMDD or severe mood dysregulation; not primary MDD trial. | 12-17 years; female by arm: IPT-MBD 7/10; TAU 1/9 (total 8/19). | IPT-MBD n=10 vs TAU n=9. | 24 weeks. | Clinical global impression severity/improvement; irritability and mood measures. | Excluded from quantitative synthesis. | PDF-verified. Table 1 reports female counts 7/10 and 1/9. Excluded because the population was DMDD/severe mood dysregulation rather than primary MDD. |
| 18 | Rice 2014 / YoDA-F; protocol paper | Protocol for young people with MDD; mixed adolescent-young adult sample. | 15-25 years; planned N=400; sex NR because protocol. | Omega-3 PUFAs + cognitive behavioural case management vs placebo + case management. | 12 weeks planned. | Depressive symptoms planned; protocol only. | Excluded from quantitative synthesis. | Protocol paper with no results; mixed young adult age range. |
| 19 | Stasiak 2012/2014; pilot randomized placebo-controlled cCBT trial | Adolescents with symptoms of depression referred by school counsellors; DSM/ICD MDD not verified for primary analysis. | Mean age 15.2 years; female 14/34 (41%); arms cCBT female 9/17, CPE female 5/17. | Computerized CBT (The Journey) n=17 vs computer psychoeducation/attention placebo n=17. | Post-intervention and 1-month follow-up. | CDRS-R primary; RADS-2 and quality-of-life/coping measures. | Excluded from quantitative synthesis. | Depressive symptoms/low mood rather than verified DSM/ICD primary MDD; not retained for primary NMA. |
| 20 | Grudin 2022; randomized feasibility trial | Adolescents with mild-to-moderate major depression. | 13-17 years; female total 19/32 (59%); by arm: therapist-guided I-BA 6/11, self-guided I-BA 7/10, TAU 6/11. | Therapist-guided I-BA n=11; self-guided I-BA n=10; TAU n=11. | 10-week intervention; primary endpoint 3-month follow-up. | CDRS-R primary; feasibility/acceptability/safety. | Disconnected direct evidence and CDRS-R-only MD sensitivity. | PDF-verified. Table 2 reports total female 19/32 and arm-level female counts 6/11, 7/10, and 6/11. Not connected to pill-placebo primary pharmacotherapy network; summarized separately. |
| 21 | TADS 2004; randomized controlled trial | Adolescents with DSM-IV MDD. | 12-17 years; mean 14.6 years; 45.6% male (54.4% female). | Fluoxetine+CBT n=107; fluoxetine n=109; CBT n=111; placebo n=112. | 12 weeks acute stage. | CDRS-R total score; CGI improvement; suicidality. | Adjusted endpoint sensitivity evidence; not primary. | Source-located values were adjusted/model-derived rather than raw arm-level endpoint mean/SD; kept separate. |
| 22 | Israel 2012; pilot randomized clinical trial | Clinic-referred adolescents with depression; ABFT feasibility in Norway. | 13-17 years; girls 11/20 (55%). | ABFT n=11 vs TAU n=9. | Pilot outpatient intervention; exact endpoint NR in abstract. | Depression symptoms; effect size reported. | Excluded from quantitative synthesis. | PDF-verified. Results, Final participants paragraph states that over half (55%) of the participants were girls. Pilot ABFT vs TAU; participant overlap with later Waraan-related reports could not be verified. |
| 23 | Radecki 2024 LVM-MD-11; phase 3 randomized double-blind study | Adolescents with MDD. | 12-17 years; female by arm: LVM 40 mg 94/134; LVM 80 mg 87/138; fluoxetine 84/134; placebo 98/141. | Levomilnacipran 40 mg n=134; levomilnacipran 80 mg n=138; fluoxetine 20 mg n=134; placebo n=141. | 8-week endpoint within randomized study. | CDRS-R primary; CGI-S secondary. | Primary SMD, CDRS-R-only MD sensitivity, model-based sensitivity. | PDF-verified. Female counts are from Table 1 safety population. Note that efficacy/ITT denominators in the endpoint table may differ for some arms (e.g., placebo n=140). Adolescent-only LVM-MD-11 retained; observed week-8 endpoint mean/SD source verified. |
| 24 | Radecki 2024 LVM-MD-14; phase 3 randomized double-blind study | Mixed children/adolescents with MDD. | 7-17 years; female by arm: LVM 40-80 mg 99/166; fluoxetine 105/166; placebo 114/160. | Levomilnacipran 40-80 mg n=166; fluoxetine 20 mg n=166; placebo n=160. | 8-week endpoint. | CDRS-R primary; CGI-S secondary. | Excluded from quantitative synthesis. | PDF-verified. Female counts are from Table 1 safety population. Efficacy/ITT denominator differs in Table 2 (LVM n=162, fluoxetine n=166, placebo n=159). Mixed 7-17 sample; no separate adolescent-only efficacy data retained. |
| 25 | Findling 2022; randomized placebo-controlled fluoxetine-referenced fixed-dose study | Adolescents with DSM-5 MDD who did not respond to BPI plus placebo lead-in. | 12-17 years; female by treated/demographic arm: vortioxetine 10 mg 93/147; vortioxetine 20 mg 96/161; fluoxetine 103/153; placebo 105/154. | Allocated/randomized: vortioxetine 10 mg n=147; vortioxetine 20 mg n=162; fluoxetine 20 mg n=153; placebo n=154. Demographic Table 1 treated N: 147, 161, 153, and 154, respectively. | 8-week double-blind period after 4-week lead-in. | CDRS-R primary; CGI-S; CGAS; safety. | Model-based sensitivity evidence. | PDF-verified. Female counts are from Table 1 treated/demographic denominator. Vortioxetine 20 mg has randomized n=162, demographic/treated N=161, and FAS denominator differs; denominator type is therefore explicitly stated. Model-based MMRM treatment differences were used only in sensitivity evidence. |
| 26 | Wagner 2003; pooled report of two RCTs | Children/adolescents with DSM-IV MDD; pooled pediatric sample. | 6-17 years; adolescents 199/376; female 108/189 sertraline and 84/187 placebo. | Sertraline 50-200 mg/day n=189 vs placebo n=187. | 10 weeks. | CDRS-R total score; response; adverse events. | Excluded from quantitative synthesis. | Pooled pediatric 6-17 trials; no adolescent-only randomized arm-level endpoint data retained. |
| 27 | Wagner 2006; randomized double-blind placebo-controlled trial | Pediatric MDD; total sample not adolescent-only; adolescent findings post hoc. | 6-17 years; female 68/131 escitalopram and 69/133 placebo. | Escitalopram 10-20 mg/day n=131 vs placebo n=133. | 8 weeks. | CDRS-R primary; safety/suicidality. | Excluded from quantitative synthesis. | Pediatric 6-17 trial; adolescent-only values post hoc and not randomized arm-level main data. |
| 28 | Waraan 2021; randomized trial | Adolescents with MDD in community mental health clinics. | 13-18 years; mean 14.9 years; female 52/60 (86.7%). | ABFT n=30 vs TAU n=30. | 16-week randomized treatment; clinician-rated post-treatment endpoint at 16 weeks, with self-report assessments during treatment. | HAMD primary; BDI-II repeated self-report. | Narrative evidence only. | PDF-verified. MDD diagnosis confirmed; randomized treatment was 16 weeks, beyond the prespecified <=12-week acute window. No eligible <=12-week arm-specific endpoint mean/SD was retained for primary analysis. |
| 29 | Weihs 2018; randomized double-blind fluoxetine-referenced trial | Children/adolescents with DSM-IV-TR MDD; mixed 7-17 sample. | 7-17 years; adolescents n=209; female 127/209 among adolescents; overall female 184/339. | Placebo, desvenlafaxine 25-50 mg/day, fluoxetine 20 mg/day; safety population total n=339. | 8 weeks. | CDRS-R primary; CGI-S/CGI-I; C-SSRS. | Excluded from quantitative synthesis. | Mixed child-adolescent failed trial; no adolescent-only endpoint mean/SD retained. |
| 30 | Wright 2017; feasibility randomized controlled trial | Adolescents with low mood/depression; DSM/ICD MDD not verified for primary analysis. | 12-18 years; female by arm derived from Table 1 male counts: Stressbusters 33/45; self-help websites 27/46. | Stressbusters CCBT n=45 vs self-help websites n=46. | 4-month outcome after intervention. | BDI primary; MFQ, SCAS, EQ-5D-Y, HUI-2. | Excluded from quantitative synthesis. | PDF-verified. Table 1 reports male counts 12/45 and 19/46; female counts were derived as 33/45 and 27/46 under the binary gender categories reported. Low mood/depression sample; MDD diagnosis and usable primary-analysis outcomes not verified. |
| 31 | Young 2016; secondary outcome report from Omega-3 + PEP pilot RCT | Youth with DSM-IV-TR depressive disorders; secondary behavioral-problem outcomes. | 7-14 years; 56.9% male (43.1% female). | PEP+omega-3, PEP+placebo, omega-3, placebo; total N=72. | 12 weeks. | SNAP-IV, ECBI behavioral outcomes; depression parent trial measures in Fristad report. | Excluded from quantitative synthesis. | Same parent Omega-3 + PEP pilot RCT as Fristad 2016; secondary behavioral outcomes, not separate parent trial. |
| 32 | Simeon 1990; placebo-controlled double-blind fluoxetine study | Adolescents with unipolar major depression. | 13-18 years; 22 female/40. | Fluoxetine vs placebo; 40 randomized/participated; 15 completers per group. | 7-week double-blind fluoxetine-placebo trial after a 1-week single-blind placebo lead-in; the abstract describes 15 completers per group in the 8-week study including lead-in. | HAM-D, CGI, Raskin, Covi Anxiety, SCL-58. | Narrative evidence only. | PDF-verified. Methods state a 1-week single-blind placebo lead-in followed by a 7-week double-blind fluoxetine-placebo trial; no source-located arm-level endpoint mean/SD or contrast SE was retained from the available article text. |
| 33 | Klein 1998; randomized placebo-controlled desipramine trial | Adolescents with MDD after single-blind placebo lead-in. | 13-18 years randomized; randomized sample female 30/45 (67%). | Desipramine n=23 vs placebo n=22 randomized; completer endpoint values used in primary SMD. | 6 weeks after 2-week placebo lead-in. | HAM-D primary endpoint used; BDI secondary. | Primary SMD and binary direct sensitivity. | Source-verified completer endpoint; reported pooled/common SD; high-risk/data-quality flag retained. |
| 34 | Rohde 2004; randomized psychosocial trial | Adolescents with MDD and conduct disorder recruited from juvenile justice setting. | 13-17 years; female 27/45 CWD-A and 18/48 life-skills/tutoring; total 45/93 (48.4%). | CWD-A n=45 vs life skills/tutoring control n=48. | 16 two-hour group sessions conducted over 8 weeks; post-treatment assessment and 6- and 12-month follow-up. | MDD recovery/remission, BDI-II, HAM-D, social functioning. | Binary direct sensitivity; disconnected direct evidence. | PDF-verified. Treatment consisted of 16 two-hour sessions over an 8-week period; not connected to the pill-placebo network, so binary/direct evidence was summarized separately. |
| 35 | Berard 2006; international multicenter placebo-controlled paroxetine trial | Adolescents with DSM-IV unipolar major depression. | Eligible adolescents were 13-18 years; mITT Table 1 reports arm-specific age ranges: paroxetine 12-19 years (mean 15.5) and placebo 13-18 years (mean 15.8); mITT female 122/182 paroxetine and 61/93 placebo. | Paroxetine n=182 vs placebo n=93 in mITT; 286 randomized total. | 12 weeks. | MADRS response; K-SADS-L depression subscale; CGI, BDI, MFQ. | Model-based or binary sensitivity evidence. | PDF-verified. Table 1 reports mITT arm-specific age ranges and female counts; retained only as model-based/binary sensitivity where applicable, not as primary raw endpoint mean/SD evidence. |
| 36 | Diamond 2002; randomized ABFT treatment-development study | Adolescents with DSM-III-R MDD. | 13-17 years; 25/32 female (78%). | ABFT vs minimal-contact waitlist; total n=32. | 12-week ABFT vs 6-week minimal-contact waitlist; follow-up reported. | MDD diagnostic status; depressive/anxiety symptoms; family conflict. | Disconnected direct or narrative evidence. | Not connected to pill-placebo primary network; not duplicate with Norway ABFT studies. |
| 37 | Mufson 1999; controlled clinical trial of IPT-A | Clinic-referred adolescents with DSM-III-R MDD. | 12-18 years; female 70.8% in clinical monitoring and 75.0% in IPT-A. | IPT-A n=24 vs clinical monitoring n=24. | 12 weeks. | HAM-D recovery criterion; social functioning and problem-solving outcomes. | Disconnected direct or narrative evidence. | Not connected to pill-placebo primary network; summarized separately. |
| 38 | Keller 2001 / Study 329; randomized controlled trial | Adolescents with DSM-IV major depression. | 12-18 years; female 58/93 paroxetine, 56/95 imipramine, 57/87 placebo. | Paroxetine n=93; imipramine n=95; placebo n=87. | 8 weeks. | HAM-D endpoint response/change; K-SADS-L; CGI. | Model-based sensitivity evidence. | Adjusted/model-based estimates only; interpreted cautiously because of RIAT/selective-reporting concerns. |

Source-verification note. Denominator type is explicitly stated where randomized, safety, treated/demographic, FAS, and efficacy/ITT denominators differ. Fields that could not be reliably verified from the available source reports are marked as NR.

**Supplementary Figures S1–S6**

**Supplementary Figure S1.** Study selection and strict source-verification classification. The upper flow reports the legacy broad-analysis screening counts; the lower flow separately reconciles the 37 source documents and 38 trial/report classification entries documented in Supplementary Table S1.

**Supplementary Figure S2.** Antidepressant SMD network. Nodes represent antidepressants and pill placebo in the source-verified endpoint mean/SD network, and edges represent direct randomized comparisons.

**Supplementary Figure S3.** CDRS-R-only connected MD sensitivity analysis. Mean differences are shown versus pill placebo; negative values favor active treatment.

**Supplementary Figure S4.** CDRS-R-only disconnected direct evidence. Mean differences are shown for direct comparisons not connected to the pill-placebo reference network.

**Supplementary Figure S5.** Model-based active-versus-placebo direct sensitivity evidence. Model-based least-squares mean or adjusted contrast estimates are shown by source scale and are not interpreted as common-scale pooled effects.

**Supplementary Figure S6.** Binary direct sensitivity evidence. Response or remission outcomes are summarized as direct evidence only because outcome definitions and network components were heterogeneous or single-study.
